# Supplementary material for: Flash glucose monitoring in young people with type 1 diabetes—a qualitative study of young people, parents and health professionals: ‘It makes life much easier’
Source: BMJ Open. 2023 Apr 19;13(4):e070477. doi: 10.1136/bmjopen-2022-070477 (PMC10124239; doi:10.1136/bmjopen-2022-070477)
Supplement: Supplementary data [file bmjopen-2022-070477supp001.pdf]

Supplementary material

Families interview schedule

Aim: to explore the experiences of young people using Freestyle Libre/Libre2 sensors and their parents/carers. E.g.… how you found out about Libre, impact on day-to-day life, training received, thoughts on using Libre in the future.

Families will be contacted via their preferred contact method (phone or video call). We will discuss Libre with parents separately and without their child present where possible.

Young people/older children (YP&C) can choose whether they discuss Libre with or without their parent/carer (P/C). Younger children will be invited to discuss their experiences with their parent/carer for support. Confirmation of consent/assent to take part will be recorded verbally.

This is a guide only. Researcher to adapt depending on whether talking to (P/C), or young people/children (YP&C) Researcher to use relevant prompts and own discretion to omit questions or alter wording as appropriate.

Background

1. Clarify from eligibility questionnaire: Using Libre, finger pricking any exp of other methods of BGM? [pump, CGM]
2. How often do you/they still use finger prick blood glucose testing?
3. What did you think when you first heard about Libre?
4. How often do you go to see a doctor or nurse about your/their diabetes?

Day-to-day use

5. What are your views on Libre at the moment?
6. In what ways has Libre changed your day-to-day life? [compared to finger pricking?] [Prompts: Positives about this way of monitoring? What is challenging about this way of monitoring? Have your views changed over time? How/why]
7. Do you use a mobile phone or the reader to scan?
8. Does Libre stay on ok? [Does it ever fall off?]
9. Do you download past readings and use the data at home or just at clinical appts?
10. What would you tell other YP/C with T1D who are thinking about using Libre?
11. *P: What is challenging about being a parent/carer of a child with diabetes? *[Don't ask if YP/C present, parent/carer may struggle to answer]

Training

12. P/YP: Did you attend training to use Libre in person or virtually?
13. P/YP: Who delivered the training?
14. C: Did a doctor or nurse show you how to use Libre?
15. How did you find the training?

16. In what ways did the training help you/them understand how to use Libre?
17. Is there anything you think should be covered in training that was not?
18. Is there anything you would change about the training?

Final thoughts

19. Do you think you/they will keep using Libre? [why]
20. Is there anything else about Libre that is important to you?
21. Do you have any further comments or thoughts about what we have discussed?

Thank you for participating.

Staff focus group/interview schedule

Focus group objectives: We would like to understand how clinical teams have integrated Freestyle Libre (and Libre 2) into their day-to-day clinical practice alongside finger prick testing. E.g.… Has the way that Libre is used with families changed over time? How has Libre impacted workloads? (In clinics with families, during MDT meetings with colleagues etc).

This is a guide only. Moderators to use further prompts and probes in response to staff responses and use own discretion to omit questions or alter wording as appropriate during focus groups/interviews.

Participant introductions:

Your role? [e.g. PDSN, Consultant]

Your current responsibilities relating to Libre?

Size of clinical team?

Proportion of patients using Libre at present?

Staff thoughts on Libre

1. What are your general views on using Libre with families at the moment?

Integrated Libre into day-to-day practice

2. How have you incorporated Libre into day-to-day practice?

Ongoing use of Libre with families

3. Who provides families with initial Libre training?
4. Has the use of Libre changed any aspects of clinical practice?

How do teams assess the effectiveness of Libre

5. How do you monitor ongoing patient benefit?
6. What changes could be made to improve Libre training and support for families?

7. What changes could be made to improve Libre training and support for new clinical staff going forward?

Final questions

8. Is there anything else about Libre/Libre 2 that is important?
9. Do you have any further comments or thoughts about what we have discussed?

Thank you for participating.
